# HIF1*α* Promotes BMP9-Mediated Osteoblastic Differentiation and Vascularization by Interacting with CBFA1

**DOI:** 10.1155/2022/2475169

**Published:** 2022-10-01

**Authors:** Yuwan Li, Ziming Liu, Hong-De Wang, Jun Zhang, Miaoyuan Lin, Jianye Yang, Jiaxing Huang, Wenqiang Yan, Yingfang Ao

**Affiliations:** ^1^Department of Sports Medicine, Peking University Third Hospital, Institute of Sports Medicine of Peking University, Beijing Key Laboratory of Sports Injuries, Beijing 100191, China; ^2^Department of Orthopaedics, The First Affiliated Hospital of Chongqing Medical University, Chongqing 400016, China; ^3^Department of Orthopaedics, The First Affiliated Hospital of Zunyi Medical University, Zunyi, 563000 Guizhou, China

## Abstract

Bone morphogenetic protein 9 (BMP9) as the most potent osteogenic molecule which initiates the differentiation of stem cells into the osteoblast lineage and regulates angiogenesis, remains unclear how BMP9-regulated angiogenic signaling is coupled to the osteogenic pathway. Hypoxia-inducible factor 1*α* (HIF1*α*) is critical for vascularization and osteogenic differentiation and the CBFA1, known as runt-related transcription factor 2 (Runx2) which plays a regulatory role in osteogenesis. This study investigated the combined effect of HIF1*α* and Runx2 on BMP9-induced osteogenic and angiogenic differentiation of the immortalized mouse embryonic fibroblasts (iMEFs). The effect of HIF1*α* and Runx2 on the osteogenic and angiogenic differentiation of iMEFs was evaluated. The relationship between HIF1*α*- and Runx2-mediated angiogenesis during BMP9-regulated osteogenic differentiation of iMEFs was evaluated by ChIP assays. We demonstrated that exogenous expression of HIF1*α* and Runx2 is coupled to potentiate BMP9-induced osteogenic and angiogenic differentiation both *in vitro* and animal model. Chromatin immunoprecipitation assays (ChIP) showed that Runx2 is a downstream target of HIF1*α* that regulates BMP9-mediated osteogenesis and angiogenic differentiation. Our findings reveal that HIF1*α* immediately regulates Runx2 and may originate an essential regulatory thread to harmonize osteogenic and angiogenic differentiation in iMEFs, and this coupling between HIF1*α* and Runx2 is essential for bone healing.

## 1. Introduction

Mesenchymal progenitor cells (MPCs) exhibit self-renewal capacity and rapidly proliferate and differentiate into multiple lineages including osteogenic, chondrogenic, myogenic, and adipogenic cell lines [1–4]. Mouse embryonic fibroblasts (MEFs) are mesenchymal stem cells that could be able to undergo multidirectional differentiation potential which is into osteogenic, chondrogenic, and angiogenic cell lines [5]. Immortalized mouse embryonic fibroblasts (iMEFs) could be capable of conditionally immortalized MEFs that possess increased proliferative ability and long-term maintenance of cell proliferation [6]. iMEFs can be induced to undergo phenotype reversal by Cre recombinase, in possession of most markers of MPCs and retain multiple directional differentiation potential, as they can differentiate into osteogenic and angiogenic lineages under suitable conditions [7, 8]. iMEFs can be used in regenerative medicine as effective seed cells to treat bone defects, nonunion, and osteogenic-related diseases due to their proliferation and differentiation abilities and stromal progenitor cell biology [9].

Osteogenic differentiation is a continuous cascade that recaps nearly entire of the molecular adjustments that occur throughout embryonic skeletal development [10]. Bone morphogenetic proteins (BMPs) consist of nearly 20 members that be part of the transforming growth factor beta (TGF-*β*) superfamily [11]. Recombinant human bone morphogenic proteins, including BMP4 and BMP7, have been extensively used in clinical applications for the efficient stimulation of bone formation and for treating bone defects [12]. It has been reported that BMP9 can be used for filling engineered bone and treating osseous defects. When combined with some biomaterials, the release of the growth factor can be controlled at the proper rate. BMP9 as one of the most potent BMPs, can induce osteogenic differentiation of multipotent progenitor cells [13]. The effect of BMP9-induced osteoblast differentiation is enhanced by regulating several downstream targets, such as HIF1*α* [14], Runx2 [15], Hey1 [16], and COX-2 [17]. However, the connotative mechanism involved in BMP9 mediated osteogenesis was still unclear.

Hypoxia-inducible factor 1*α* (HIF1*α*) is a generally accepted angiogenesis cascade regulator that is involved in many biological and organic developmental processes, including tumor-related and skeletal development. Some studies also indicated that HIF1*α* helps promote BMP9-induced angiogenic and osteogenic differentiation though BMP/Smad signaling in iMEFs. Runx2 is a specific transcription factor in mature bone cells that plays an important role in the formation and reconstruction of bone tissue. Runx2 regulates the differentiation of iMEFs into osteoblasts and promotes the maturation and vascularization of cartilage. Additionally, this protein is involved in the synthesis of extracellular matrix. However, it is currently unclear whether and how the angiogenic factor HIF1*α* is coupled with the osteogenic factor Runx2 during BMP9-induced osteogenesis in iMEFs. It is also ambiguous whether these factors mutually participate in this regulation.

In this study, we investigated the role of HIF1*α* and Runx2 on BMP9-induced angiogenic and osteogenic differentiation in MEFs. We found that exogenous overexpression of HIF1*α* and Runx2 intensifies BMP9-induced osteogenic and angiogenic differentiation of iMEFs *in vitro* and in animal model, whereas RNA-mediated silencing of HIF1*α* and Runx2 strongly dulls BMP9-induced angiogenic and osteogenic signaling in iMEFs. Mechanistically, HIF1*α* can directly regulate Runx2 expression at the transcript and protein levels. Our findings no mere broad our understanding of the molecular events underlying BMP9-induced osteoblast-specific differentiation but also suggest that targeting the coupling of BMP9-induced angiogenic and osteogenic signaling may become a new approach for vascular-targeted therapy in bone tissue engineering.

## 2. Materials and Methods

### 2.1. Cell Culture and Chemicals

The experiment was approved by the Ethics Committee of the First Affiliated Hospital of Chongqing Medical University, and the written informed consent was obtained before surgery. HEK-293 cells and iMEFs were obtained from ATCC (Manassas, VA, USA). Cells were maintained in low-glucose Dulbecco's modified Eagle's medium (LG-DMEM) containing 10% fetal bovine serum (FBS), 100 U/mL penicillin, and 100 mg/mL streptomycin. The cells were incubated at 37°C with 5% CO_2_. The cultured medium was changed every three to four days.

### 2.2. Recombinant Adenovirus Arrangement

The recombinant adenoviruses were constructed with AdEasy technology [18, 19]. To be short, the core regions of genes encoding BMP9, HIF1*α*, green fluorescent protein (GFP), red fluorescent protein (RFP), and Runx2 were amplified by quantitative real-time reverse transcription polymerase chain reaction (qRT–PCR), cloned into shuttle vectors which could be subjected to amplify recombinant adenoviruses in HEK-293 cells. The target sites of siRNA against mouse HIF1*α* and Runx2 coding regions were amplified into the pSES adenoviral shuttle vector which form recombinant adenoviruses. The adenoviruses nominated Ad-BMP9, Ad-HIF1*α*, Ad-Runx2, Ad-Sim-HIF1*α*, and Ad-Sim-Runx2. Ad-BMP9 expressed GFP, while Ad-HIF1*α*, Ad-Sim-HIF1*α*, Ad-Runx2, and Ad-Sim-Runx2 expressed RFP as a visual sign for observing efficiency of infection. Analogous adenoviruses expressing monomeric GFP (Ad-GFP) served as negative controls.

### 2.3. Gene Expression of Osteogenesis- and Angiogenesis-Related mRNA

The cells were cultured according to the experimental design. Total RNA was gained with TRIzol reaction agentia, and cDNA was extracted from RNA with a reverse transcription reaction reagent (RR047A, TAKARA, Japan). The first-strand cDNA products were diluted 5-10-fold and used as templates for detection by real-time qRT-PCR (RR820A, TAKARA). The amplification conditions were set: predenaturation, denaturation, and annealing. GAPDH served as the internal control set. Expression levels of target genes from various groups were measured using the 2^ΔΔCT^ method. Gene expression of collected samples was conducted triplicate. The information of primers for PCR are shown in Table 1.

### 2.4. Chromatin Immunoprecipitation (ChIP) Assay

Subconfluent iMEFs were treated with infection of Ad-GFP or Ad-BMP9 and Ad-HIF1*α*. The cell samples were cross-linked after 48 hours of infection, then subjected to ChIP analysis according to the manufacturer's instructions (#9003, CST). The cells were incubated with a rabbit anti-mouse HIF1*α* (HIF1*α*67, Abcam) antibody or IgG to pull down the DNA-protein Mmixture. The Runx2 promoter sequence was determined using four pairs of primers consistent with the mouse Runx2 sites. The relevant primers and sequences of promoter information of the ChIP assay are listed in Supplementary Tables 1 and 2.

### 2.5. Alkaline Phosphatase (ALP) Expression Assay

ALP expression was measured by a modified automated chemiluminescence assay (BD Company, USA) and histochemical staining, according to the manufacturers' instruction [20, 21]. Cells were treated with infection of Ad-GFP, Ad-HIF1*α*, Ad-BMP9, Ad-Runx2, and Ad-BMP9 combined with Ad-HIF1*α*, Ad-BMP9 combined with Ad-Runx2, Ad-BMP9 combined with Ad-SimHIF1*α*, or Ad-BMP9 combined with Ad-Sim-Runx2. In the chemiluminescence detection and quantification test, each sample was tested for three times, and the tests were repeated in at least three independent experiments. In addition, ALP expression was standardized to the total cellular protein level of cell samples. ALP expression was presented as the mean ± standard deviation.

### 2.6. Matrix Mineralization Detection

iMEFs were inoculated in 24-well plates for 6 h seeded at a density of 30% and then infected with Ad-BMP9, Ad-HIF1*α*, or Ad-Runx2. The osteoblastic differentiation-conditioned medium contained ascorbic acid and *β*-phosphoglycerol for 21 days. The matrix mineralization activity was evaluated by Alizarin Red S staining. The cell samples were soaked with 2.5% glutaraldehyde 15 min at room temperature, washed with PBS solution for three times. The mineralized tubercles were stained with 0.4% (*v*/*v*) Alizarin Red S dyestuff for 10 min, then washed with double distilled water for 15 min. Mineralization was observed under a microscope, and at least 3 independent experiments were conducted.

### 2.7. Nano-Glo Dual-Luciferase Reporter Assay

The iMEFs were seeded in Corning 24-well cell culture plates and transfected with 1 *μ*g firefly luciferase vector, 1 *μ*g Renilla luciferase vector, and 0.5 *μ*g BMP9 binding element luciferase reporter (p12Xsbe-Luc) or Runx2 reporter plasmid (p6Xose2-Luc) per flask using Lipofectamine 2000 [22, 23]. Twenty-four hours after transfection, the cells were reseeded in 24-well plates and treated with DMSO, HIF1*α*, and/or Runx2. After 36 h, the cell samples were lysed and subjected to a luciferase assay using a Promega Luciferase assay kit (E1500; Promega, Madison, WI, USA). Luciferase activities were normalized by detecting the concentration of total cellular protein.

### 2.8. Immunohistochemical Staining

Cells were infected with adenovirus and treated in accordance with the experimental design. The iMEFs were seeded onto sterile sections in 12-well plates at a density of 10^4^ cells/mL. The cells were washed 3 times with PBS for 10 min, then the cells were fixed with 4% paraformaldehyde at 37°C for 15 min, washed 3 times with PBS for 10 min, and permeabilized using 0.3% Triton X-100 for 30 minutes at 37°C. The cell samples were blocked with goat serum for 30 minutes after permeabilization. The samples were then incubated with primary antibodies overnight. The results of the expected protein were visualized by incubation with the appropriate fluorescently labeled secondary antibody. The sections were carefully removed and then mounted on slides with glycerol.

### 2.9. Stem Cell Implantation and Ectopic Ossification

iMEFs were transfected with appointed adenoviruses and prepared for injection at 5 × 10^6^ cells into the side abdomen of athymic nude mice (4-7-week-old male, Sprague–Dawley) until fluorescence could be seen. Five weeks after injection, the mice were euthanized, and bony masses were obtained for micro-CT imaging and histologic staining and evaluation.

### 2.10. Microcomputed Tomographic Imaging Analysis

The bone masses were retrieved and scanned with SkyScan1174 X-ray microtomography (micro-CT) (Bruker Company, Belgium) after the animals were euthanized at 5 weeks. The 3D image reconstruction was analyzed with N-Recon software, and all image data analysis was performed using CT-SCAN software. Histomorphological parameters of bone formation including bone volume/total volume (BV/TV), trabecular number (Tb.N), trabecular separation (Tb.Sp), trabecular thickness (Tb.Th), and bone mineral density were detected.

### 2.11. Hematoxylin and Eosin (H&E) and Trichrome Staining

The bone mass samples were collected and decalcified with 10% EDTA solution, then soaked in PBS for three times and fixed in 4% paraformaldehyde. The samples embedded in paraffin were subjected to H&E and Trichrome Masson's staining in accordance with manufacturers' instructions [24, 25].

### 2.12. HUVEC Tube Formation Assay

HUVECs were seeded in the enhanced endothelial conditioned medium (ECM), which is containing 10% FBS, 2 mM L-glutamine, 100 U/mL penicillin, 100 *μ*g/mL streptomycin, and 1% ECGS (Sigma, USA) for 4 h. The HUVECs were transferred into lower storey of the Transwell which is the preparation of Matrigel at a density of 10^5^ cells per well. Then, the experimental iMEFs transfected with adenovirus were transferred into the upper wells. The Transwell was incubated at 37°C for 4 h. The tube area was quantified with the relative area of tube structures formed. The relative area of tubes was counted for three times per well and averaged from three images per well using Image J software (National Institutes of Health, USA).

### 2.13. Subcutaneous PLGA-iMEF Hybrid Implantation to Detect Angiogenesis

iMEFs were transfected with specific adenovirus in accordance with the study design for 24 h and transferred onto poly (lactic-co-glycolic acid) (PLGA) scaffolds. Eighteen mice (6-week-old males; BALB/cAnN, Chongqing, China) weighing 18-25 g were anesthetized with 1% pentobarbital sodium (30 mg/kg), and then, PLGA scaffolds carrying transfected iMEFs were implanted into the subcutaneous region of nude mice. At the end of the fifth week, the implants were harvested. The PLGA-iMEF composites were collected and analyzed after 5 weeks. The PLGA scaffold-iMEF composites were retrieved. The samples were, respectively, decalcified and paraffin-embedded, then the sections were performed with immunohistochemical staining for CD31 (Abcam, USA), then incubated with the fluorophore-conjugated antibodies.

### 2.14. Statistical Analysis

The results were assessed; the data represented correspond to the mean ± SD if not stated. Experiment was conducted three times *n* = 3. Statistical analyses were performed using GraphPad Prism app (CA, USA). Student-Newman-Keuls *T* tests and one-way ANOVA were used to analyze the significant differences from various groups. Level of significance was determined as *p* < 0.05 significance.

## 3. Results

### 3.1. HIF1*α* and Runx2 Promote BMP9-Mediated Osteoblastic Differentiation of iMEFs

**Figure 1:**
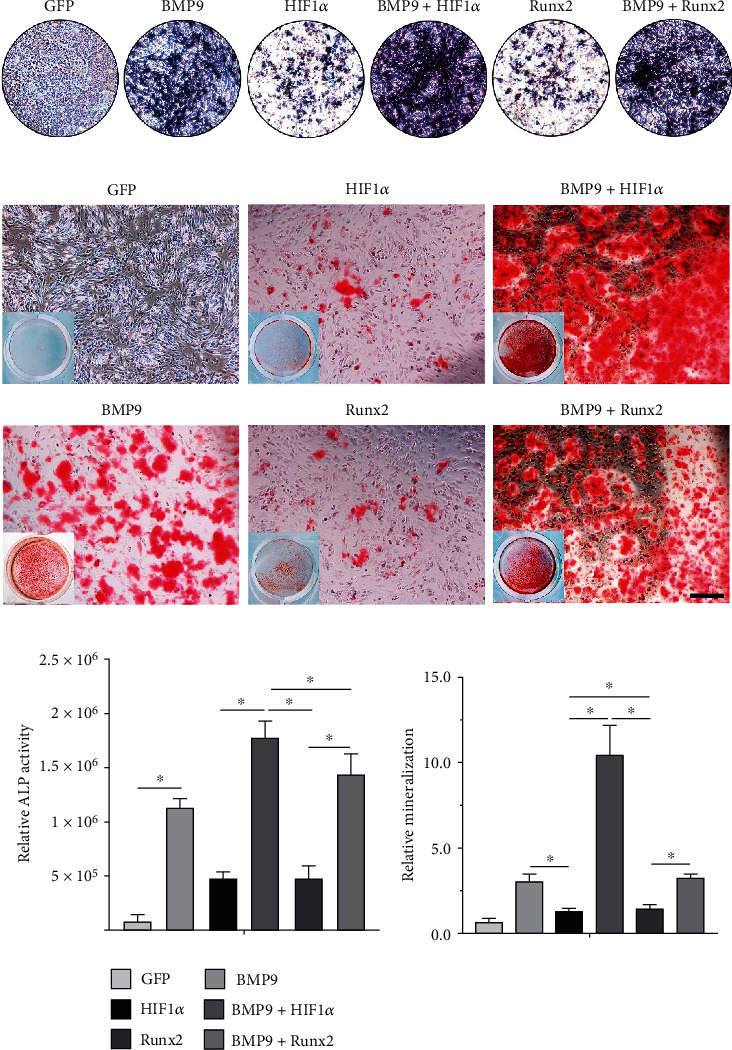
HIF1*α* and Runx2 potentiate BMP9-induced early osteogenic differentiation and late matrix mineralization of iMEFs. (a) Overexpression of HIF1*α* promotes BMP9-induced alkaline phosphatase (ALP) activity in iMEFs. (b) An Alizarin Red S staining assay was used to evaluate the matrix mineralization of iMEFs. Representative gross images and microscopic images in response to the above treatments are shown after transfection. Alizarin Red S staining shows the effect of HIF1*α* and Runx2 on BMP9-induced matrix mineralization in iMEFs (scale bar = 100 *μ*m). (c) Quantification results showed that HIF1*α* and Runx2 enhanced BMP9-induced ALP activity and matrix mineralization, respectively (^∗^*p* < 0.05).

**Figure 2:**
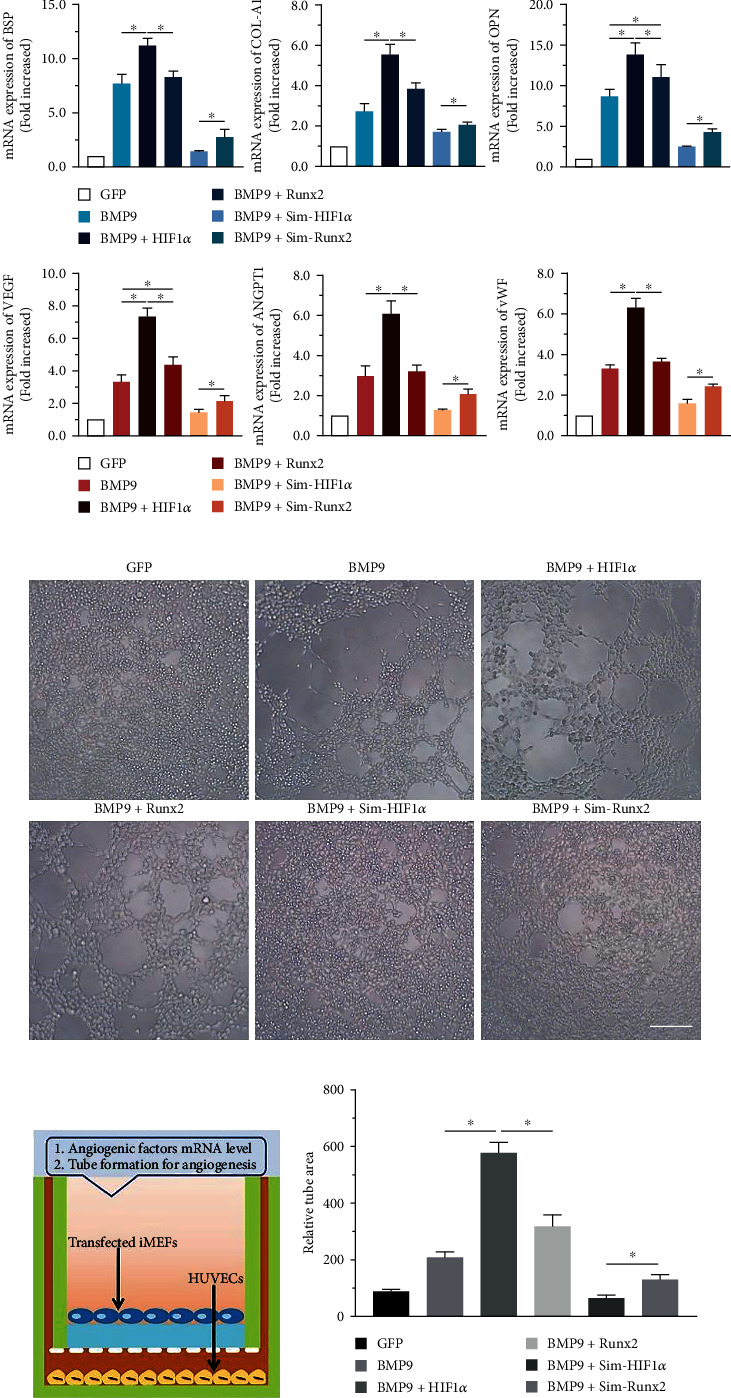
Effects of HIF1*α* and Runx2 on BMP9-induced early osteogenic and angiogenic differentiation in iMEFs. (a) Effect of mRNA expression of HIF1*α* and Runx2 on BMP9-induced osteogenic and angiogenic regulators, including VEGF, ANGPT1, and vWF, on day 7 (^∗^*p* < 0.05). (b) Microscopy images of the effect of transfected iMEFs on tube formation by HUVECs (scale bar = 100 *μ*m). (c) Experimental design for the assay investigating the early effects on HUVEC tube formation. HUVECs were incubated in ECM medium for 12 h and plated on a Matrigel layer. iMEFs transfected with adenovirus were seeded into the upper wells. Tube formation was recorded over the course of 6 h. (d) Quantification of tube formation by HUVECs. The relative tube area was quantified to measure tube formation, and all values are shown for each group (^∗^*p* < 0.05).

To determine whether HIF1*α* and Runx2 are critical targets of BMP9-mediated osteogenic and angiogenic signaling, we sought to explore whether exogenous expression of HIF1*α* and Runx2 participates in BMP9-induced osteogenic differentiation of iMEFs. Recombinant adenoviruses expressing HIF1*α* (Ad-HIF1*α*) or Runx2 (Ad-Runx2) were constructed. The results demonstrated that HIF1*α* and Runx2 effectively mediate transgene expression in iMEFs (Supplementary Figure 1A). Alkaline phosphatase (ALP), an indicator of early-stage osteogenic differentiation, was used to detect ALP activity. The results qualitatively and quantitatively showed that HIF1*α* and Runx2 exerted an intense synergistic effect on BMP9-induced ALP activity. On day 7, HIF1*α* and Runx2 significantly enhanced BMP9-mediated ALP expression, while exogenous HIF1*α* and Runx2 alone did not produce a marked effect on the early stage of osteogenic differentiation (Figures 1(a) and 1(c)). The calcium deposition and mineralization levels of iMEFs in the BMP9+HIF1*α* and BMP9+ Runx2 groups were higher than those in the HIF1*α*, Runx2, and GFP groups on day 21 (Figures 1(b) and 1(c)). Bone sialoprotein (BSP), collagen type I (COL-A1), and osteopontin (OPN) are essential indicators of osteogenic differentiation of iMEFs. To further confirm whether HIF1*α* and Runx2 are vital mediators of BMP9-mediated osteogenesis, we investigated the role of HIF1*α* and Runx2 on the mRNA expression of genes encoding BSP, COL-A1, and OPN. The results demonstrated that the mRNA expression levels of genes encoding BSP, COL-A1, and OPN in the BMP9+HIF1*α* group were significantly upregulated compared with the levels in the BMP9 group and the HIF1*α* group at day 7. Consistent with the Runx2-mediated effect on osteogenic differentiation, the mRNA levels of these factors in the BMP9+Runx2 group were greatly increased compared to the levels in the GFP and Runx2 groups on day 7 (Figure 2(a)). Remarkably, the expression of genes encoding BSP, COL-A1, and OPN was also lower in the HIF1*α* and Runx2 groups than in the GFP group. Taken together, these results strongly indicated that HIF1*α* and Runx2 can critically potentiate the BMP9-induced early and late osteoblastic differentiation of iMEFs *in vitro*.

### 3.2. Silencing HIF1*α* and Runx2 Inhibits BMP9-Mediated Osteogenesis

**Figure 3:**
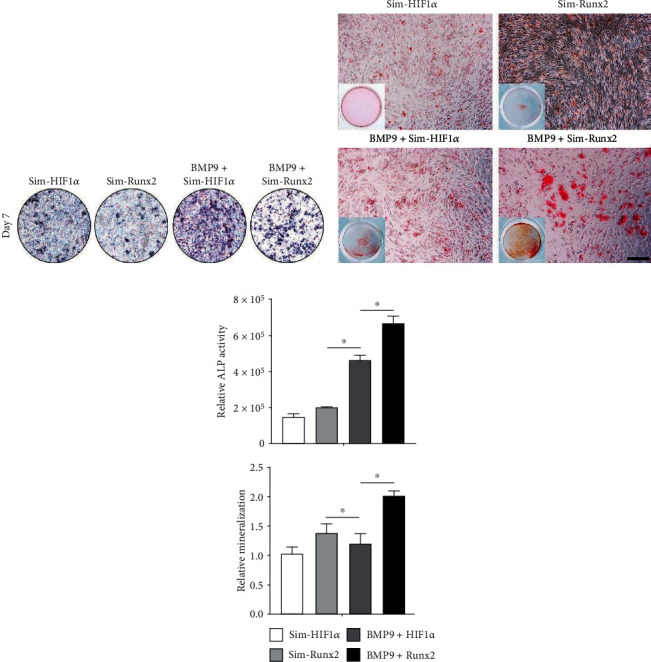
Silencing HIF1*α* and Runx2 effectively diminishes the BMP9-induced early osteogenic differentiation and late matrix mineralization of iMEFs. (a) Silencing HIF1*α* and Runx2 inhibited BMP9-induced ALP activity in iMEFs. (b) The matrix mineralization of iMEFs was evaluated by an Alizarin Red S staining assay. Representative gross images and microscopic images of cells subjected to the above treatments are shown after transfection. Alizarin Red S staining shows the effect of silencing HIF1*α* and Runx2 on BMP9-induced matrix mineralization in iMEFs (scale bar = 100 *μ*m). (c) Quantification results showed that silencing HIF1*α* and Runx2 diminished BMP9-induced ALP activity and matrix mineralization, respectively (^∗^*p* < 0.05).

We further confirmed that HIF1*α* and Runx2 are essential mediators of BMP9-induced osteogenic signaling. We used recombinant adenovirus expressing a pool of three siRNAs poniting the coding regions of mouse HIF1*α* and Runx2 using pSOS system, generating Ad-Sim-HIF1*α* and Ad-Sim-Runx2. On day 7, the results showed that compared with that of the Sim-Runx2 group, ALP activity was greatly decreased in iMEFs after transfection with Ad-Sim-HIF1*α*. Qualitatively, ALP staining results presented similar results, and the expression of Sim-HIF1*α* drastically reduced ALP activity in iMEFs induced by BMP9 (Figures 3(a) and 3(c)). Moreover, Sim-HIF1*α* expression almost totally blunted BMP9-mediated bone mineralization in iMEFs, as shown by Alizarin Red S staining and the relative mineralization rate on day 21 (Figures 3(b) and 3(c)). Similarly, the qRT–PCR results illustrated that the expression of genes encoding osteogenesis-related factors such as BSP, COL-A1, and OPN was significantly downregulated in the Sim-HIF1*α* group compared to the BMP9 group at day 7 (Figure 2(a)). In short, these results strongly demonstrated that silencing HIF1*α* and Runx2 expression may exert a negative regulatory effect on the BMP9-induced osteogenic differentiation of iMEFs.

### 3.3. HIF1*α* and Runx2 Are Critical for Junctional Differentiation of BMP9-Induced Subcutaneous Bone Formation in Nude Mice

**Figure 4:**
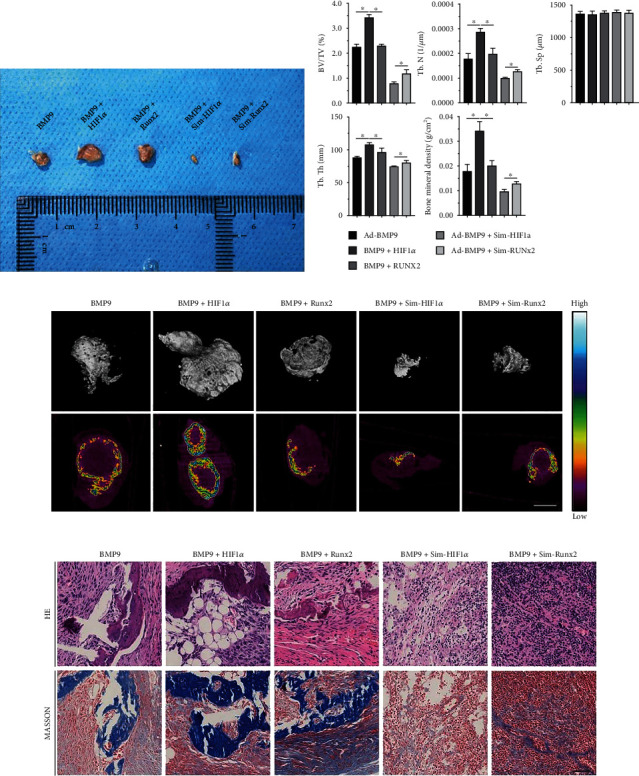
Effect of HIF1*α* and Runx2 on BMP9-induced ectopic osteogenesis after iMEF transplantation into nude mice. (a) BMP9-induced ectopic bone osteogenesis was enhanced by overexpression of HIF1*α* and Runx2 and inhibited by silencing HIF1*α* and Runx2. General observation of subcutaneous bone masses in nude mice. (b) Quantitative analysis of osteogenic masses and the relative values of BV/TV, Tb.N, Tb.Sp, Tb.Th, and bone mineral density (BMD) were analyzed (^∗^*p* < 0.05). (c) Subcutaneous osteogenic masses at 5 weeks were subjected to micro-CT analysis to investigate the 3D surface and generate a heatmap of average mineralization density. In the heatmap analysis, white represents the highest average mineral density, and black represents the lowest average mineral density (scale bar = 1 mm). (d) The retrieved samples were subjected to histologic staining, and the retrieved samples were fixed, decalcified, paraffin embedded, and subjected to H&E and Masson's trichrome staining (scale bar = 50 *μ*m).

It still remains unknown whether HIF1*α* and Runx2 could affect bone formation *in vivo*. We hence utilized a subcutaneous bone formation model to detect the effects of HIF1*α* and/or Runx2 on BMP9-induced ossification. The general morphology and micro-CT three-dimensional (3D) reconstruction results demonstrated that the general sizes of bony masses from the BMP9+HIF1*α* group were larger than those of bony masses from all other groups. The volume of osteogenic masses was smaller in the BMP9+Runx2 group than that in the BMP9+HIF1*α* group, while the BMP9+Sim-HIF1*α* group showed a distinct decrease compared with the BMP9+Sim-Runx2 group (Figure 4(a)). The average mineral density displayed by the heatmap analysis revealed that HIF1*α* and Runx2 expression potentiated the average mineralization formed by BMP9-induced iMEFs. Notably, silencing exogenic HIF1*α* and Runx2 expression robustly decreased the average mineral density, but the effect of silencing HIF1*α* was greater than the effect of silencing Runx2 (Figure 4(c)). Quantitative results of bone parameters showed that the values including bone volume/total volume (BV/TV), trabecular number (Tb.N), trabecular thickness (Tb.Th), and bone mineral density (BMD) were significantly higher in the BMP9+HIF1*α* group than in the other groups. However, the values of these parameters in the BMP9+Sim-Runx2 group were significantly higher than those in the BMP9+ Sim-HIF1*α* group. There were no significant differences in trabecular spacing (Tb.Sp) among groups (Figure 4(b)).

The retrieved bone blocks were further subjected to histologic staining. The H&E and Masson's trichrome staining results demonstrated that compared with the BMP9 group, overexpression of HIF1*α* and Runx2 significantly promoted the number and quality of trabecular bone as well as mineralization, and HIF1*α* increased these values more than Runx2 (blue in Masson's trichrome and red in H&E) among groups. Moreover, silencing HIF1*α* and Runx2 significantly decreased the amount of trabecular bone. Notably, less trabecular bone and bone matrix formation was observed in the BMP9+Sim-HIF1*α* group than in the BMP9+Sim-Runx2 group (Figure 4(d)).

### 3.4. HIF1*α* and Runx2 Regulate Angiogenic Differentiation and Vasoformation during BMP9-Mediated Osteogenesis

To further confirm the potential of angiogenic differentiation and vasoformation regulated by HIF1*α* and Runx2, we used qRT–PCR of the transfected iMEFs on day 7 and tube formation assays of HUVECs after 6 h of Transwell cultivation. The qRT–PCR assay revealed that the mRNA expression of genes encoding angiopoietin 1 (ANGPT1), vascular endothelial growth factor (VEGF), and von Willebrand factor (vWF) was significantly upregulated in the BMP9+HIF1*α* group compared with the other groups (*p* < 0.05). It is worth noting that the mRNA expression of ANGPT1, VEGF, and vWF was higher in the BMP9+Sim-Runx2 group than in the BMP9+ Sim-HIF1*α* group (Figure 2(a)).

The phenotypes of isolated HUVECs at the third passage were identified, and they highly expressed CD31, VEGF, EMCN, and vWF (Supplementary Figure 2). Tube formation assays showed that HIF1*α* significantly increased the relative tube area. Runx2 also increased the tube area, albeit to a lower degree than HIF1*α*, both independently and in combination with BMP9. Moreover, the relative tube area in the BMP9+Sim-HIF1*α* group was greatly decreased compared with that in the BMP9+ Sim-Runx2 group (Figures 2(b)–2(d)). Taken together, these data indicate that HIF1*α* and Runx2 are able to jointly regulate BMP9-induced angiogenic signaling and vascularization, and Runx2 may be an essential downstream target of HIF1*α* that participates in angiogenesis.

### 3.5. Effects of HIF1*α* and Runx2 on BMP9-Induced Vessel Invasion

**Figure 5:**
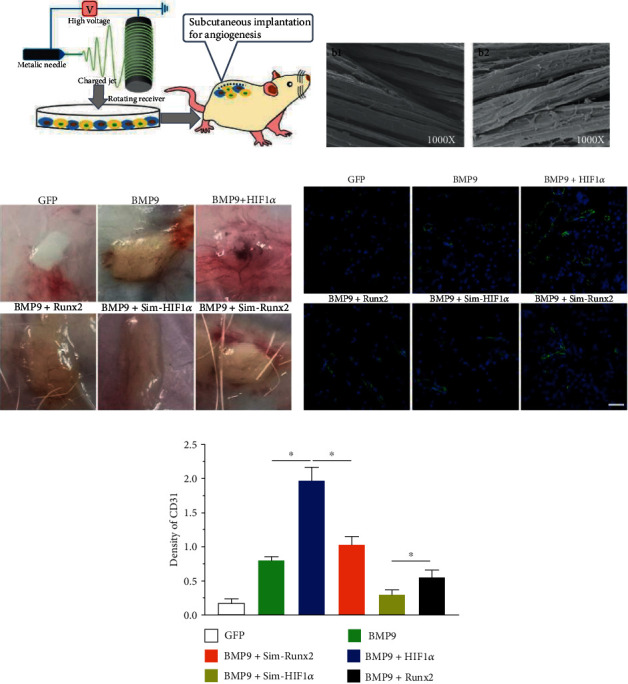
*In vivo* effects of the PLGA-transfected iMEF composite on vessel formation upon subcutaneous implantation into rats. (a) Schematic diagram showing the transfected iMEFs seeded on electrospun PLGA scaffolds implanted into the dorsal subcutaneous space of the mice. (b) Scanning electron microscopy image of the PLGA scaffold (b1) and seeded cells (b2) on the PLGA scaffold (magnification = 1000x). (c) General observation of harvested PLGA implants at 5 weeks. (d) Immunofluorescence staining for CD31 (vascular formation displays green fluorescence) and the average number of vascular structures in a high-power field for each implant (scale bar = 50 *μ*m). (e) Quantification in each group is based on immunofluorescence staining.

To further investigate the effect of HIF1*α* and Runx2 on BMP9-induced angiogenesis *in vivo*, iMEFs were transfected with the appropriate adenovirus, seeded on PLGA scaffolds, and implanted in the dorsal subcutaneous tissue of male rats (Figure 5(a)). The cells adhered well to the PLGA scaffold after seeding for 72 h (Figure 5(b)). After gross observation of vascular invasion to the implants, PLGA scaffolds exhibited a vascularization effect, among which the vascularization ability in the BMP9+HIF1*α* group was more apparent (Figure 5(c)). The implant histology demonstrated that PLGA scaffolds seeded with cells in the BMP9+HIF1*α* group showed greater levels of CD31 (Figure 5(d)). Remarkably, CD31 expression in the BMP9+Sim-Runx2 group was higher than that in the BMP9+ Sim-HIF1*α* group (Figure 5(e)). In summary, HIF1*α* significantly potentiated BMP9-induced angiogenesis *in vivo*, and silencing HIF1*α* inhibited the angiogenesis of iMEFs.

### 3.6. Runx2 Is Directly Upregulated by HIF1*α* in BMP9-Stimulated iMEFs

**Figure 6:**
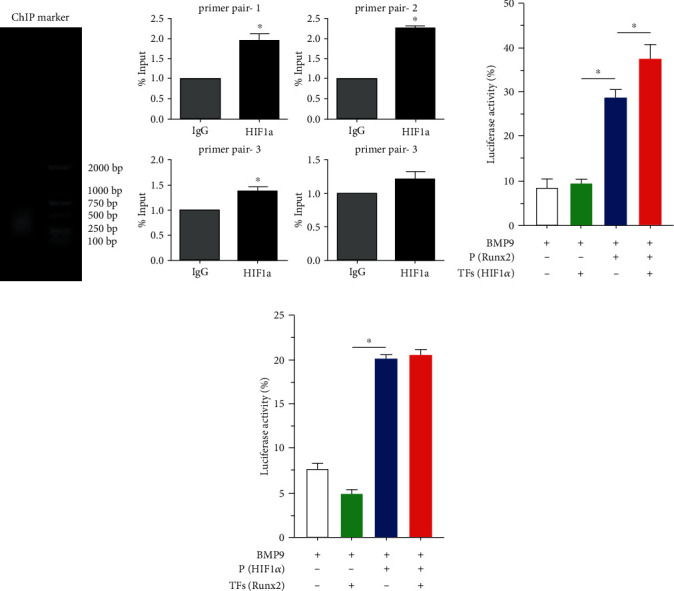
Identification and verification of Runx2 as an early downstream target of HIF1*α* enhanced by BMP9 in iMEFs. (a) ChIP analysis showed that Runx2 is a direct target of HIF1*α* signaling in iMEFs. iMEFs were transfected with Ad-BMP9 and Ad-HIF1*α* for 36 h, followed by formaldehyde crosslinking. The crosslinked cells were lysed and subjected to enzymolysis and immunoprecipitation with an anti-HIF1*α* or IgG antibody, the pulled down composite was subjected to gel electrophoresis and imaging, and primers for the Runx2 promoter region were used in the ChIP assay. (b) The recovered chromatin DNA fragments were used for qPCR amplification with 4 primers specific for the mouse Runx2 promoter. (c) Luciferase reporter assay results showed the effect of HIF1*α* and/or Runx2 on the transcriptional activities of the firefly/Renilla luminescence reporter. (d) The transcription factor Runx2 cannot activate the promoter of HIF1*α*. The transcription factor HIF1*α* can activate the promoter of Runx2 (^∗^*p* < 0.05).

Our experiments confirmed that HIF1*α* and Runx2 are essential for osteoblastic differentiation of BMP9-stimulated iMEFs; therefore, we sought to determine whether HIF1*α* exerts any impact on BMP9-induced Runx2 expression. We used a ChIP assay to determine the relationship between HIF1*α* and Runx2 in BMP9 signaling. Gel electrophoresis was used to analyze the pulled down composite using an anti-HIF1*α* antibody, and the observed length was between 100 and 200 bp (Figure 6(a)). The location of promoter-specific primers for Runx2 yielded the expected products upon transfection with BMP9+HIF1*α*. We found pairs of primers targeting Runx2 that amplified the expected fragments in the group transfected with BMP9+HIF1*α* (Figure 6(b)). We further determined the coupling effect between HIF1*α* and Runx2 at the transcription level by using a luciferase reporter assay. The results indicated that the transcriptional activity of the Runx2 luciferase reporter was significantly upregulated by HIF1*α* upon BMP9 stimulation for 48 hours (Figure 6(c)). However, the transcriptional activity of the HIF1*α* luciferase reporter gene was not obviously different upon Runx2 expression (Figure 6(d)). These data indicated that the transcription factor HIF1*α* can activate the promoter of Runx2 to regulate the initiation and activation of Runx2 at the gene transcription level, and the osteogenic differentiation induced by HIF1*α* and Runx2 may also be mediated by the activation of the BMP9-induced signaling pathway in iMEFs.

## 4. Discussion

HIF1*α* is a well-established and vital regulator of angiogenesis, and it is necessary to elicit angiogenic signals to recruit new blood vessels and maintain many developmental processes, including skeletal development [26–28]. Runx2 is among the most vital transcription factors, is indispensable for osteogenic differentiation, and is responsible for the activation of osteogenesis marker genes [29, 30]. However, it remains unclear how HIF1*α* is coupled with Runx2 during the process of BMP9-induced osteogenic differentiation and bone formation. In this study, we explored whether HIF1*α* directly regulated Runx2 and whether HIF1*α*-mediated angiogenesis affected BMP9-stimulated osteogenic differentiation of MPCs. We detected the effect of HIF1*α* and Runx2 on ALP activity as an early osteogenic factor, bone mineralization as a late osteogenic indicator, the expression of osteogenesis- and angiogenesis-related factors and consolidated subcutaneous ectopic bone formation of iMEFs in athymic nude mice, increased osteogenic volume, and the values of BV/TV, Tb.Th, Tb.N, and BMD; we also investigated HUVEC tube formation in prevascular-like structures after *in vitro* and *in vivo* PLGA subcutaneous implantation for angiopoiesis. Overall, the results demonstrated that the exogenous expression of HIF1*α* and Runx2 enhances BMP9-stimulated osteogenic differentiation, including ALP activity, calcium deposition, and the expression of osteogenesis-related factors such as BSP, COL-A1, OPN, and ectopic subcutaneous bone formation, yet silencing HIF1*α* and/or Runx2 strongly blocked BMP9-induced osteogenic signaling in iMEFs. Furthermore, while stable overexpression of HIF1*α* and Runx2 can potentiate the angiogenesis-related factors ANGPT1, VEGF, and vWF and support HUVEC orientation into prevascular-like structures *in vitro* and intensify robust vascularization in ectopic subcutaneous implantation of PLGA-transfected iMEFs, silencing HIF1*α* and Runx2 can profoundly inhibit these effects *in vitro* and *in vivo*. Mechanistically, HIF1*α* can directly regulate the activation of Runx2 at the transcriptional level, and HIF1*α* also exerts a synergistic effect by promoting angiogenic and osteogenic signaling pathways upon BMP9 induction in iMEFs. Therefore, our results strongly demonstrate that the coupling between HIF1*α* and Runx2 plays an essential role in BMP9-induced osteogenic and angiogenic differentiation.

HIF1*α* is a well-established factor that regulates adaptive responses, including capillary ingrowth and angiogenesis, to reduce oxygen utilization, both spatially and temporally. It was previously reported that oxygen-sensitive HIF*α* subunits are found in mammals: HIF1*α*, HIF2*α*, and HIF3*α*. HIF1*α* and HIF2*α* have been widely studied and are the most common [31]. There are two regulatory models of HIF1*α*. Under normoxia, HIF1*α* is rapidly hydroxylated and ubiquitinated by the E3 ubiquitin ligase complex, which contains the von Hippel-Lindau disease tumor suppressor (VHL) protein and undergoes rapid degradation. Another regulatory model of HIF1*α* is that HIF1*α* is hydroxylated at asparagine residues by inhibiting the HIF1 factor to prevent the transcriptional activity of HIF1*α*, which is accomplished by blocking the interaction of the transcriptional coactivator cAMP response element binding protein (CBP) and histone acetyltransferase p 300 (P 300 HAT) with HIF1*α* [32, 33]. In contrast, under hypoxic conditions, HIF1*α* is stabilized by limited oxygen as a helper substrate for prolyl hydroxylase domain enzymes (PHDs), and the HIF1*α* protein hydroxylation rate is reduced by enhancing the transcriptional activation of PHD, HIF1, and CBP-p 300 coactivated complexes. Then, HIF*α* levels increase, and HIF1*α* target genes are expressed [34, 35]. Recent studies have shown that stimulating angiogenesis plays a key role in the process of increased bone mass while the HIF1*α*-VEGF pathway is activated [33]. Some research found that VEGF enhances BMP-mediated stimulation of anabolic bone formation by affecting angiogenesis [36–38]. The possible nonautonomous mechanisms underlying the overexpression of HIF1*α* in mature mouse osteoblasts include the possibility that blocking the VHL protein significantly enhances angiogenesis and osteogenesis, including the activation of VEGF in vascular endothelial cells. Hence, in mice lacking HIF-1*α*, the trabecular bone volume and bone formation rate were significantly reduced, the cortical bone structure was changed, and vascular development in the long bones was decreased [39]. Our study showed that overexpression of exogenous HIF1*α* intensified BMP9-induced osteogenic differentiation and osteogenesis. Angiogenesis-related factors (e.g., VEGF, ANGPT1, CD31, and vWF) were upregulated, and the activity of prevascular structures and subcutaneous vasoformation were higher in the HIF1*α*-expressing group.

In contrast, silencing HIF1*α* impaired the osteoblast and angiogenic differentiation ability, as observed in the cells treated with Sim-HIF1*α*. Therefore, HIF1*α* signaling in anabolic bone formation may increase osteogenesis via a cell-nonautonomous effect to integrate spatiotemporal expansion and revascularization and establishment of the blood supply in bone, although it does not preclude the existence of additional effectors. Moreover, we believe that HIF1*α* signaling is a critical accommodator of new vessels for bone regeneration.

Runx2 belongs to the small transcription factor family, and members of the Runx family contain the runt domain [40]. Runx2 is a vital regulator in bone development and is essential for osteoblast differentiation. Mutation of Runx2 is related to skeletal malformation syndromes, including cleidocranial dysplasia (CCD) [41, 42]. Runx2 knockout mice lose all intramembranous and endochondral osteogenesis and die after birth due to lack of mineralization in the chest region, which results in breathing difficulties, even in the presence of BMP [43]. The DNA sequence 5′-PuACCPuCA-3′ and its complementary sequence 5′-TGPyGGTPy-3′ are recognized by Runx2, which is capable of binding to the promoters of genes encoding type I collagen, osteopontin, osteocalcin, BSP, collagenase-3, VEGF, type X collagen, and others to enhance the expression of these proteins and boost the osteoblastic differentiation [44–47]. These results may indicate that Runx2 is important for regulating osteogenesis, chondrocyte hypertrophy, and vascular invasion of the developing skeleton [15, 48]. Our results revealed that Runx2 overexpression is capable of promoting BMP9-induced early and late osteogenesis and ectopic subcutaneous bone formation and upregulating the expression of osteogenesis- and angiogenesis-related genes. In addition, Runx2 organizes HUVECs into prevascular-like structures *in vitro* and promotes vascularization *in vivo* after PLGA implantation, even though Runx2-mediated osteogenesis and angiogenesis were less robust than those induced by HIF1*α*. Conversely, silencing Runx2 attenuated the effects of BMP9-stimulated osteogenesis and angiogenesis, further confirming that Runx2 is a key regulator of osteoblastic and angiopoietic differentiation and a molecular transducer in osteogenic biology.

Even though bone formation is primarily mediated by osteoblasts, many other tissues in bone consist of vascular endothelium and/or sensory and motor nerves that help establish a conducive milieu for bone formation [49–54]. Our *in vitro* HUVEC tube formation assays and *in vivo* analysis of angiogenesis after PLGA implantation showed that the level of CD31 was increased by overexpression of HIF1*α* and Runx2. Furthermore, the expression of angiogenic-related factors was higher after overexpressing HIF1*α* than that elicited by Runx2, and silencing HIF1*α* decreased the expression of these factors to a greater degree than did inhibiting Runx2.

**Figure 7:**
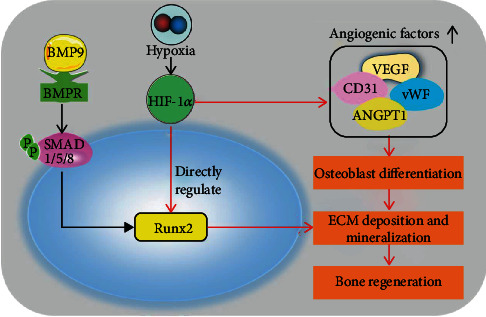
Proposed regulatory loop involving BMP9, HIF1*α*, and Runx2 in iMEFs. Runx2 and osteogenic marker factors are upregulated by BMP9 through BMP/Smad1/5/8 signaling to promote osteogenesis. A hypoxic environment leads to elevated HIF1*α* levels, which directly regulate the transcriptional function of Runx2 in the nucleus. Runx2 is an important target of HIF1*α* in iMEFs that regulates osteogenic differentiation *in vitro* and *in vivo*. Another possible important branch of the regulatory loop between HIF1*α* and osteogenesis is that HIF1*α* is a vital factor that plays a positive role in vascularization by regulating the expression of vasoformation-related regulators, including VEGF, CD31, vWF, and ANGPT1. Our initial results support the notion that the transcript activity of Runx2 is regulated by and coupled to HIF1*α*, which is enhanced by BMP9. This loop not only strengthens the osteogenic differentiation of iMEFs and angiogenesis mediated by HIF1*α* but also enhances extracellular matrix deposition and mineralization, which boosts efficient bone regeneration.

We further evaluated the relationship between HIF1*α* and Runx2 at the gene level. Luciferase reporter assays showed that the transcription factor HIF1*α* was able to enhance luciferase expression downstream of the Runx2 promoter. The mRNA of Runx2 was shown to be pulled down with an anti-HIF1*α* antibody. Mechanistically, these data demonstrated that Runx2 is a downstream target of HIF1*α* and that HIF1*α* can directly regulate Runx2 (Figure 7).

## 5. Conclusion

In summary, this study recognized the ability of HIF1*α* and Runx2 to stimulate osteogenic and angiogenic differentiation of iMEFs and showed that silencing HIF1*α* and Runx2 inhibited BMP signaling. The coupling effect of HIF1*α* and Runx2 may play an essential role in osteogenic and angiogenic pathways during the process of bone formation in BMP9-induced iMEFs. In our study, only adenoviruses were used to upregulate and downregulate exogenous expression of HIF1*α* and Runx2, but in clinical applications, adenoviruses must meet a higher standard. It is possible to use adenoviruses in animal models, but they have not yet been used in clinical applications. Additionally, the exact mechanism by which HIF1*α* and Runx2 regulate BMP9-induced effects in iMEFs and vascular endothelial cells remains unclear and needs further study.

## Figures and Tables

**Table 1:** Primer sequence of the target genes.

Gene	Forward primer (5′-3′)	Reverse primer (5′-3′)	Product length
BMP9	TACAACAGATACACAACGGACA	GATGTTGAAGATCAGGATGTGC	135
Runx2	CAGACCAGCAGCACTCCATATC	CCGTCAGCGTCAACACCATC	182
HIF1*α*	TGCTCATCAGTTGCCACTTCC	TGCCTTCATCTCATCTTCACTGTC	139
OPN	GACCGTCACTGCTAGTACACAAG	CCTTAGACTCACCGCTCTTCATG	194
BSP	AAGCACAGACTTTTGAGTTAGC	ACTTCTGCTTCTTCGTTCTCAT	145
COL-A1	TGAACGTGGTGTACAAGGTC	CCATCTTTACCAGGAGAACCAT	234
VEGF	CACGACAGAAGGAGAGCAGAAG	CTCAATCGGACGGCAGTAGC	82
ANGPT1	TTCTTCGCTGCCATTCTGACTCAC	GTTGTACTGCTCTGTCGCACTCTC	165
vWF	ACAGTAACATGGAGATGGCAGTG	TTGTGGCGTGTATGTGAGGATG	143

## Data Availability

All data generated or analyzed during this study are included in this published article (and its supplementary information files).

## Abbreviations

AHR: Aryl-hydrocarbon receptor
ANGPT1: Angiopoietin 1
BMD: Bone mineral density
BMP9: Bone morphogenetic protein 9
BMPs: Bone morphogenetic proteins
BMSCs: Bone marrow stromal cells
BSP: Bone sialoprotein
CBP: cAMP response element-binding protein
CCD: Cleidocranial dysplasia
ChIP: Chromatin immunoprecipitation assays
COL-A1: Collagen type I
COX-2: Cytochrome c oxidase subunit II
ECM: Endothelial conditioned medium
EMCN: Endomucin
FBS: Fetal bovine serum
GFP: Green fluorescent protein
H&E: Hematoxylin and eosin
Hey1: Hes-related family bHLH transcription factor with YRPW motif 1
HIF1*α*: Hypoxia-inducible factor 1*α*
HUVECs: Human umbilical vein endothelial cells
iMEF: Immortalized mouse embryonic fibroblast
MPCs: Mesenchymal progenitor cells
OPN: Osteopontin
PHD: Prolyl hydroxylase domain enzymes
PLGA: Poly(lactic-co-glycolic acid
qRT-PCR: Quantitative reverse transcription polymerase chain reaction
RFP: Red fluorescent protein
Runx2: Runt-related transcription factor 2
TGF-*β*: Transforming growth factor beta
VEGF: Vascular endothelial growth factor
VHL: von Hippel-Lindau disease tumor suppressor
vWF: von Willebrand factor.

## Supplementary Materials

Supplementary Materials Supplementary Figure 1 The recombinant adenovirus Ad-GFP (green), Ad-BMP9 (green), Ad-HIF1*α* (red), Ad-Sim-HIF1*α* (red), Ad-Runx2 (red), and Ad-Sim-Runx2 (red) were effectively transfected into iMEFs as observed by fluorescence microscopy (scale bar = 100 *μ*m). Supplementary Figure 2: phenotype and identification of HUVECs. Immunofluorescence staining assay of the typical biomarkers of CD31, VEGF, EMCN, and vWF of HUVECs (scale bar = 100 *μ*m). Supplementary Table 1: oligonucleotides used for ChIP amplicon. Supplementary Table 2: the promoter sequence of Runx2.
