# Supplementary material for: HIF1α Promotes BMP9-Mediated Osteoblastic Differentiation and Vascularization by Interacting with CBFA1
Source: Biomed Res Int. 2022 Oct 1;2022:2475169. doi: 10.1155/2022/2475169 (PMC9547689; doi:10.1155/2022/2475169)
Supplement: Supplementary Materials — Supplementary Figure 1 The recombinant adenovirus Ad-GFP (green), Ad-BMP9 (green), Ad-HIF1α (red), Ad-Sim-HIF1α (red), Ad-Runx2 (red), and Ad-Sim-Runx2 (red) were effectively transfected into iMEFs as observed by fluorescence microscopy (scale bar = 100 μm). Supplementary Figure 2: phenotype and identification of HUVECs. Immunofluorescence staining assay of the typical biomarkers of CD31, VEGF, EMCN, and vWF of HUVECs (scale bar = 100 μm). Supplementary Table 1: oligonucleotides used for ChIP amplicon. Supplementary Table 2: the promoter sequence of Runx2. [file 2475169.f1.docx]

Supplementary materials

**HIF1α promotes BMP9-mediated osteoblastic differentiation and vascularization by interacting with CBFA1**


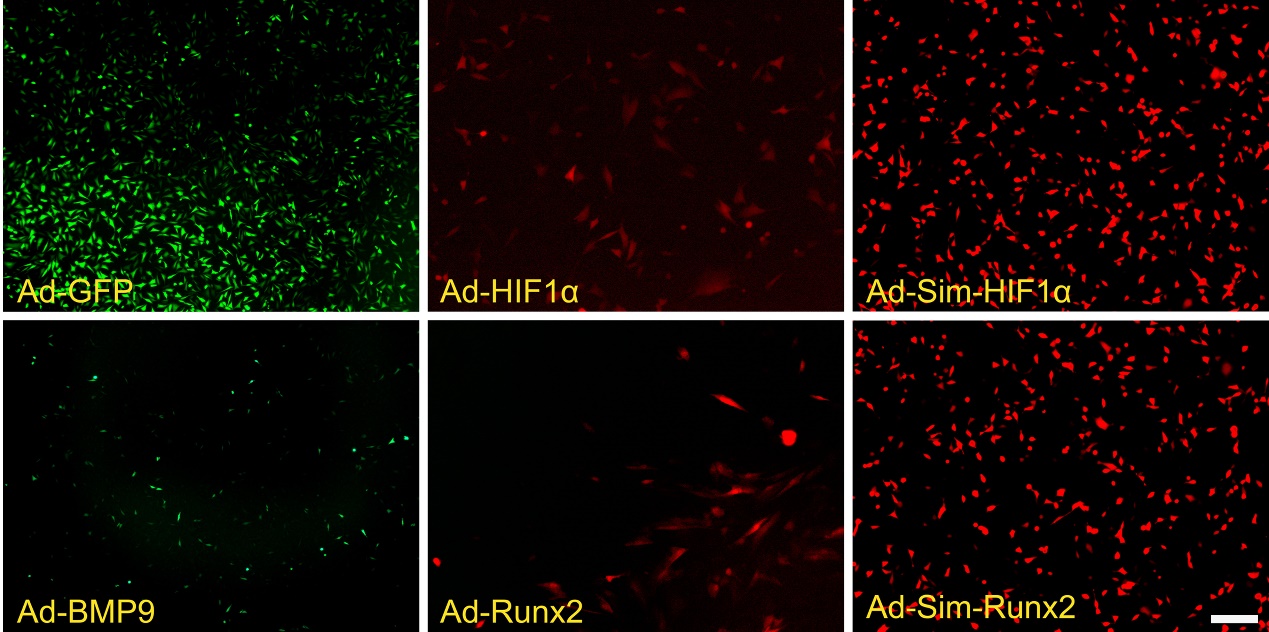


**Supplementary Fig 1.** The recombinant adenovirus Ad-GFP (green), Ad-BMP9 (green), Ad-HIF1α (red), Ad-Sim-HIF1α (red), Ad-Runx2 (red) and Ad-Sim-Runx2 (red), were effectively transfected into iMEFs as observed by fluorescence microscopy (Scale bar=100μm).


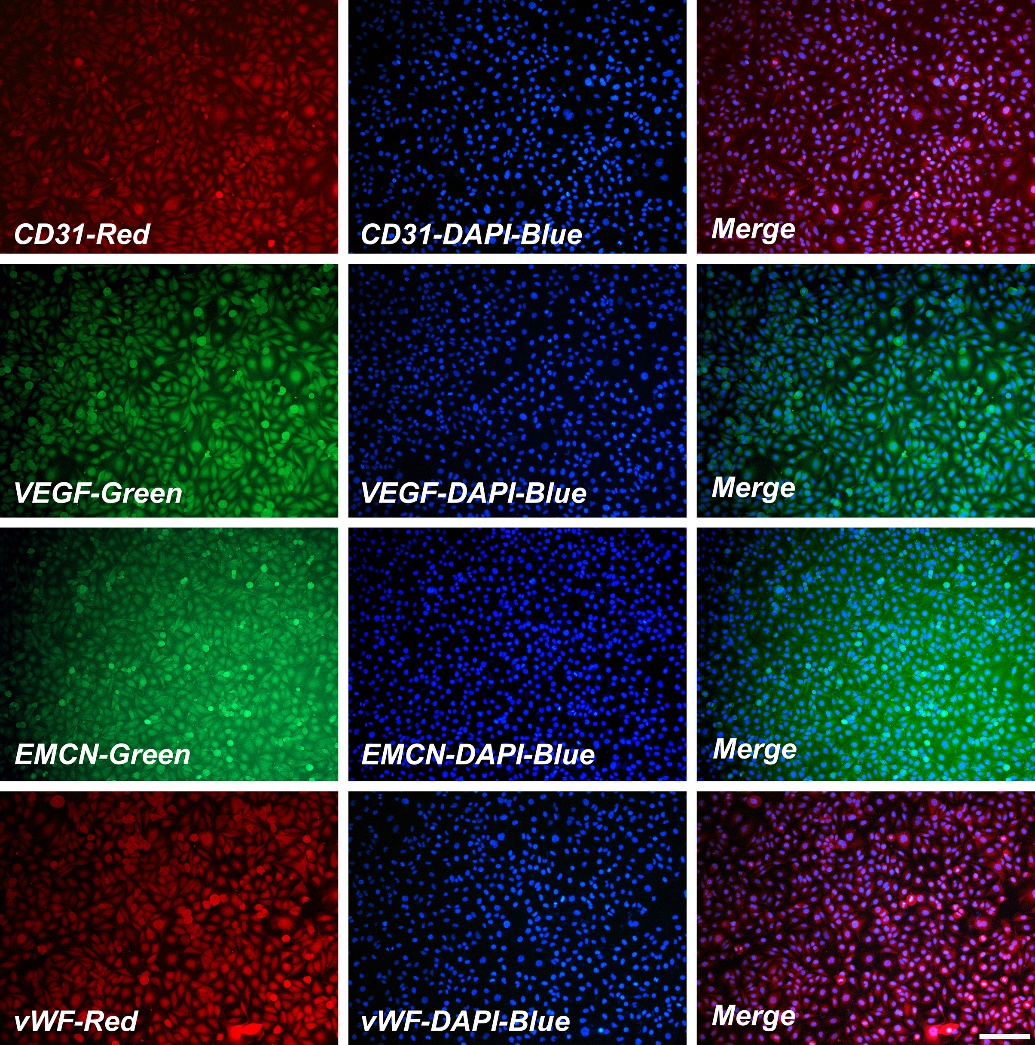


**Supplementary Fig 2.** Phenotype and identification of HUVECs. Immunofluorescence staining assay of the typical biomarkers of CD31, VEGF, EMCN, and vWF of HUVECs (Scale bar= 100 μm).

**Supplementary Table 1**. Oligonucleotides used for ChIP amplicon.

| Gene | Species | Accession No. | Primer Sequence |
| --- | --- | --- | --- |
| PP-1 | Mouse | [NM_001145920.2](https://www.ncbi.nlm.nih.gov/nuccore/NM_001145920.2) | CACCATCACAGTCATCCGTTCCATGCCACTCCTGGTTACCATCACACTAGGAAGAAATCTAACATGCAAATTCAGAGTGGCGTGGATAAATGGCAAAAAATGCCTAGGAAATTGGTCTGCTCGCCTTTA |
| PP-1 | Mouse | genomic | Forward primer (5'-3') CACCATCACAGTCATCCGTTCC  Reverse primer (5'-3') TAAAGGCGAGCAGACCAATTTCC |
| PP-2 | Mouse | [NM_001145920.2](https://www.ncbi.nlm.nih.gov/nuccore/NM_001145920.2) | GATACAATCCCAAGATGCGAATTACTGCAAAGCAGCACTGTTGCTCAGAACGCCACACACTCAGTTGAGACAATTTTGCTCACTTTTCCATAGACATAATAATGAAGGAAAGGGAGGAGGGGTAGAGAAGAGAGATGAAAAAGCAGAGGAGGGAAGGGGGAGTAGGGAGGTGGCAGAAAG |
| PP-2 | Mouse | genomic | Forward primer (5'-3') GATACAATCCCAAGATGCGAATTACTG  Reverse primer (5'-3') CTTTCTGCCACCTCCCTACTCC |
| PP-3 | Mouse | [NM_001145920.2](https://www.ncbi.nlm.nih.gov/nuccore/NM_001145920.2) | AGGGAGAGGACAAGAGAAGAGAAAGGAGGGAGGGGAGGGGAGAAGGAAAAAGATTGAGAAAGAGGGAGGGAAGAGAGCAAGGGGGAAGCCACAGTGGTAGGCAGTCCCACTTTACTTTG |
| PP-3 | Mouse | genomic | Forward primer (5'-3') AGGGAGAGGACAAGAGAAGAGAAAG  Reverse primer (5'-3') CAAAGTAAAGTGGGACTGCCTACC |
| PP-4 | Mouse | [NM_001145920.2](https://www.ncbi.nlm.nih.gov/nuccore/NM_001145920.2) | GCAAATCAAGACGACTAACATACTCTGTCTGTGTGCATTATTCCTTACTACACACAGCATTT |
| PP-4 | Mouse | genomic | Forward primer (5'-3') GCAAATCAAGACGACTAACATACTCTG  Reverse primer (5'-3') AAATGCTGTGTGTAGTAAGGAATAATGC |

**Supplementary Table 2.** The promoter sequence of Runx2

| **>5' Flanking sequence chromosome:GRCm38:17:44814798:44816797:-1** |
| --- |
| ATATGGAACTAAGTTCAGAAACTCCACAAATTATATAGACAAAACCCTTTTTTTATTTACTTTGAATAATAGAGATAAAGATCACACTGGCACACTTTATTTATGAAAGAGGATAATAGAGTAACTTTTTTCTCCTCTGCATGAATAATGACCCTAAATGAAAACTTCAGTATAAATATCTGTTTTACAGTAAAACATGAGTCTAGCCTCAAAAATCAAACAAAAGAATGTATTTCTGTGGTTTTGTCATTAAAACTTTATTCTGAAAAATTAAATAAATAAACCTAGATTCTTGAAAAATAAGGGGTTAAAAGCATTACCATGTCTTTCCAGTATATAGAGAATAAATGTTTAAAGAATCTTATGAACATGATTTCATAGATAACTTTAACTAAGAGGAAACAAAAACAGACAATGAGTTATTTTGGGGTGTACAGACACAAGAATATTTTACTTCTGTCACCCTCTAAGTCACTCCCTCTTACCTCCACTGTGCACCCCAAATAATTTCTTGTACTTCTGTGCCCCCACCCACCATCACAGTCATCCGTTCCATGCCACTCCTGGTTACCATCACACTAGGAAGAAATCTAACATGCAAATTCAGAGTGGCGTGGATAAATGGCAAAAAATGCCTAGGAAATTGGTCTGCTCGCCTTTATAATGTTTGTTGAAAAATCCTCCATCGCTCCCAACTAATGAAAACAGGAAGCTCTATTCATAAATGTGAAATTCACTGCCTATGATATATAATCATCCTAATAAGAAAATGAGCTCTAGACATACATGTCCAAGAGGGCAAAAGAAGAGATAGTTTCCCAAAGATGGTTTCAATTTTCTTCTGAATCAGAATTAGCAAATCAAGACGACTAACATACTCTGTCTGTGTGCATTATTCCTTACTACACACAGCATTTTGTAATTTATTTCAAAGCTTCCATTATAAACAACAAAAACTTACAGTTTCTGTTAACCCCCTCTATTCTGAGCTATGGAAATTACTGCATATTTCATTATATATGCAGAACTGCACCCAAAGTCCTGTTACAGTCACTGTCCACGCTGATGAAAGAATTATACAAAACATTTCTTTGAAAGATAAAATCCAATCATACAGAAAACTAACATTAGTCCAACAAAATGTCCACCACAATTCCTGACATTTGTTTTTTAAGATCTTCAAAGTAACCATGGGATGATGGCAAAAATAATGTAAACGATACTAATTACATTTAATCTTTATTGTAAGAGCCGCCACGTAATAAAAAAAAAAAAATCAACTACACAGCCATGATTTAATATTTGTAAAGGAATCCCCAGGCTAACACTTTTGTGACAGCCAATTACAGTCGATCCCGATCCCGGCAAGGAGTTTGCAAGCAGAGCTCTGGAAAGGTAAACTCCTTTTTACAATGAGTTACAGATCCCCAAGCTTAGGAAGACAAGCAAAAGGCAAACAGAAGGAAGCAGCCACCCTGGGAAATCCGAAGCAGCCTTGCAAGTGATACAATCCCAAGATGCGAATTACTGCAAAGCAGCACTGTTGCTCAGAACGCCACACACTCAGTTGAGACAATTTTGCTCACTTTTCCATAGACATAATAATGAAGGAAAGGGAGGAGGGGTAGAGAAGAGAGATGAAAAAGCAGAGGAGGGAAGGGGGAGTAGGGAGGTGGCAGAAAGGAAAAGCCTTAGCTACAGAGTTCTGCTCTCCAGAGGCTTAACCTTACAGGAGTGTGGGCTCCTTCAGCATTTGTGTTCTAGCCAAATCCTCATGAGTCACAAAAATTAAAAAGCTATAACCTTCTGAATGCCAGGAAGGCCTTACCACAAGCCTTTTGTCAGAGGGAGAAAGGGAGAGAGAGAGGGAGAGAGAGAGGGAGAGAGAGAGGGAGAGAGGAAGGGAAGGAGAGACAGAGGAACACCCATAAGTAAAGAGACAGAAGGAAGGAAAGGGAGAGGACAAGAGAAGAGAAAGGAGGGAGGGGAGGGGAGAAGGA |
